# Receipt of Telehealth Services, Receipt and Retention of Medications for Opioid Use Disorder, and Medically Treated Overdose Among Medicare Beneficiaries Before and During the COVID-19 Pandemic

**DOI:** 10.1001/jamapsychiatry.2022.2284

**Published:** 2022-08-31

**Authors:** Christopher M. Jones, Carla Shoff, Kevin Hodges, Carlos Blanco, Jan L. Losby, Shari M. Ling, Wilson M. Compton

**Affiliations:** 1 National Center for Injury Prevention and Control, US Centers for Disease Control & Prevention, Atlanta, Georgia; 2 Information Products and Analytics Group, US Centers for Medicare & Medicaid Services, Baltimore, Maryland; 3 National Institute on Drug Abuse, National Institutes of Health, Bethesda, Maryland; 4 US Centers for Medicare & Medicaid Services, Baltimore, Maryland

## Abstract

**Importance:**

Federal emergency authorities were invoked during the COVID-19 pandemic to expand use of telehealth for new and continued care, including provision of medications for opioid use disorder (MOUD).

**Objective:**

To examine receipt of telehealth services, MOUD (methadone, buprenorphine, and extended-release [ER] naltrexone) receipt and retention, and medically treated overdose before and during the COVID-19 pandemic.

**Design, Setting, and Participants:**

This exploratory longitudinal cohort study used data from the US Centers for Medicare & Medicaid Services from September 2018 to February 2021. Two cohorts (before COVID-19 pandemic from September 2018 to February 2020 and during COVID-19 pandemic from September 2019 to February 2021) of Medicare fee-for-service beneficiaries 18 years and older with an *International Statistical Classification of Diseases, Tenth Revision, Clinical Modification* OUD diagnosis.

**Exposures:**

Pre–COVID-19 pandemic vs COVID-19 pandemic cohort demographic characteristics, medical and substance use, and psychiatric comorbidities.

**Main Outcomes and Measures:**

Receipt and retention of MOUD, receipt of OUD and behavioral health-related telehealth services, and experiencing medically treated overdose.

**Results:**

The pre–COVID-19 pandemic cohort comprised 105 240 beneficiaries; of these, 61 152 (58.1%) were female, 71 152 (67.6%) were aged 45 to 74 years, and 82 822 (79.5%) non-Hispanic White. The COVID-19 pandemic cohort comprised 70 538 beneficiaries; of these, 40 257 (57.1%) were female, 46 793 (66.3%) were aged 45 to 74 years, and 55 510 (79.7%) were non-Hispanic White. During the study period, a larger percentage of beneficiaries in the pandemic cohort compared with the prepandemic cohort received OUD-related telehealth services (13 829 [19.6%] vs 593 [0.6%]; *P* < .001), behavioral health-related telehealth services (28 902 [41.0%] vs 1967 [1.9%]; *P* < .001), and MOUD (8854 [12.6%] vs 11 360 [10.8%]; *P* < .001). The percentage experiencing a medically treated overdose during the study period was similar (18.5% [19 491 of 105 240] in the prepandemic cohort vs 18.4% [13 004 of 70 538] in the pandemic cohort; *P* = .65). Receipt of OUD-related telehealth services in the pandemic cohort was associated with increased odds of MOUD retention (adjusted odds ratio [aOR], 1.27; 95% CI, 1.14-1.41) and lower odds of medically treated overdose (aOR, 0.67; 95% CI, 0.63-0.71). Among beneficiaries in the pandemic cohort, those receiving MOUD from opioid treatment programs only (aOR, 0.54; 95% CI, 0.47-0.63) and those receiving buprenorphine from pharmacies only (aOR, 0.91; 95% CI, 0.84-0.98) had lower odds of medically treated overdose compared with beneficiaries who did not receive MOUD.

**Conclusions and Relevance:**

Emergency authorities to expand use of telehealth and provide flexibilities for MOUD provision during the pandemic were used by Medicare beneficiaries initiating an episode of OUD-related care and were associated with improved retention in care and reduced odds of medically treated overdose. Strategies to expand provision of MOUD and increase retention in care are urgently needed.

## Key Points

**Question:**

How were federal emergency authorities to expand telehealth use for substance use disorder treatment and facilitate provision of medications for opioid use disorder (MOUD) used during the COVID-19 pandemic among Medicare beneficiaries with opioid use disorder (OUD)?

**Findings:**

In this cohort study including 175 778 beneficiaries, receipt of OUD-related telehealth services during the COVID-19 pandemic was associated with improved MOUD retention and lower odds of medically treated overdose.

**Meaning:**

Emergency authorities to expand telehealth utilization and provide MOUD flexibilities during the COVID-19 pandemic were used among Medicare beneficiaries and were associated with improved MOUD retention and lower odds of medically treated overdose, lending support for permanent adoption.

## Teaser

This cohort study examines receipt of telehealth services, medications for opioid use disorder (MOUD: methadone, buprenorphine, and extended-release naltrexone) receipt and retention, and medically treated overdose before and during the COVID-19 pandemic among Medicare beneficiaries.

## Introduction

In the US, 3 medications for opioid use disorder treatment (MOUD) are approved by the US Food and Drug Administration: methadone, buprenorphine, and extended-release (ER) naltrexone.^1^ Access to MOUD has historically been highly regulated, especially for methadone and buprenorphine, and regulatory constraints have been identified as barriers to MOUD engagement.^2^ Methadone for OUD is limited to federally certified opioid treatment programs (OTPs), with most patients required to receive methadone in person daily. Although buprenorphine can be prescribed in office-based settings, its use for OUD treatment is limited to clinicians with a Drug Addiction Treatment Act (DATA) waiver. Depending on the waiver limit, clinicians can only prescribe up to 30, 100, or 275 current patients. ER naltrexone can be prescribed by any licensed clinician operating within their scope of practice, but given costs and logistical challenges related to medication induction, it is used less often than methadone or buprenorphine.^1,2^

COVID-19 pandemic–related stressors and implementation of mitigation measures, such as stay-at-home orders, raised concerns about increasing overdose risk among individuals with OUD and disruptions to MOUD and other treatment and recovery support services.^3,4,5,6,7,8,9,10,11^ To facilitate access to care for individuals with OUD during the pandemic, multiple federal actions were initiated since declaration of the nationwide emergency on March 13, 2020.^12,13^

The US Centers for Medicare & Medicaid Services (CMS) used emergency and existing authorities to support broader use of Medicare telehealth clinical services, including through audio-only communications.^14,15^ The Substance Abuse and Mental Health Services Administration (SAMHSA) relaxed policies to significantly expand take-home doses of methadone treatment from OTPs and allowed telehealth for delivery of services at OTPs.^16^ SAMHSA and the Drug Enforcement Administration allowed DATA-waivered clinicians to remotely prescribe buprenorphine to new patients without conducting an in-person examination.^14,17^ Additionally, on January 1, 2020, CMS implemented new authorities that established a new Medicare benefit category for OUD treatment furnished by OTPs under Medicare Part B with a new bundled payment policy; this provided an additional avenue for MOUD just prior to and during the COVID-19 pandemic.^18^

Studies have documented initial findings related to how health systems,^19,20,21^ OTPs,^22,23,24^ and office-based treatment providers^25,26,27^ have leveraged these new authorities to modify service delivery during the COVID-19 pandemic. While these studies provide important insights, they largely focus on single health systems or jurisdictions (ie, state or city), how clinicians have modified their practices, or have only examined the initial months of the COVID-19 pandemic. Understanding how these authorities affect care and service use during the COVID-19 pandemic is critical to inform decisions about permanent adoption of these authorities. In response, we conducted a longitudinal cohort study using Medicare data from September 2018 to February 2021 to examine and compare the use of OUD and behavioral health-related telehealth services, receipt and retention of MOUD, and experiencing medically treated overdose among beneficiaries initiating OUD-related care before and during the pandemic.

## Methods

### Data Sources

Multiple CMS data sources were used in this study. The Medicare Beneficiary Summary File (MBSF) Base was used to identify demographic characteristics^28^; the MBSF Chronic Conditions segment^29^ and Other Chronic and Potentially Disabling Conditions segment^29^ were used to identify mental health and chronic medical conditions; Minimum Data Set 3.0 was used to determine time spent in nursing homes; and CMS claims data were used to identify substance use disorder (SUD) diagnoses, MOUD receipt, receipt of telehealth services, and experiencing medically treated overdose. The study was covered by the Common Rule exemption, 45 CFR 46.104(d)(4)(iv), and did not require institutional review board review.

### Cohort Design and Population

A retrospective cohort design was used, consisting of Medicare fee-for-service beneficiaries 18 years or older. We constructed 2 mutually exclusive cohorts of adults with OUD: a pre–COVID-19 pandemic cohort and a COVID-19 pandemic cohort. Both cohorts comprised individuals starting new (index) episodes of OUD-related care. Individuals already receiving OUD-related services, as identified by *International Statistical Classification of Diseases, Tenth Revision, Clinical Modification *(*ICD*-*10*-*CM*) diagnosis for OUD (eTable 1 in the Supplement) during the 6-month preindex period for each cohort, were excluded. The 6-month preindex period (September 2018 to February 2019 for the prepandemic cohort; September 2019 to February 2020 for the pandemic cohort) was followed by a 6-month index period (March 2019 to August 2019 for the prepandemic cohort; March 2020 to August 2020 for the pandemic cohort) when beneficiaries were enrolled in each cohort. The 6-month cohort follow-up period (September 2019 to February 2020 for the prepandemic cohort; September 2020 to February 2021 for the pandemic cohort) was chosen to align with use of 180 days as a quality measure for follow-up and retention in OUD-related care^30^ (eFigure in the Supplement). Both cohorts comprised beneficiaries with continuous fee-for-service Medicare Parts A, B, and D enrollment during their respective cohort study period or until their death from any cause following enrollment in the cohort, no preindex period *ICD*-*10*-*CM* diagnosis for OUD, and an OUD diagnosis based on at least 1 paid service claim during the cohort index period.

### Outcomes

The main outcomes were (1) receipt of MOUD (any MOUD, MOUD from OTPs, buprenorphine from pharmacies, and ER naltrexone from pharmacies) and MOUD retention; (2) receipt of telehealth services (any, behavioral health-related, and OUD-related care); and (3) experiencing medically treated overdose. Receipt of buprenorphine or ER naltrexone from pharmacies was defined as having a Part D pharmacy claim with a National Drug Code for single-entity or combination buprenorphine products approved for OUD treatment or for ER naltrexone. Receipt of methadone, buprenorphine, or ER naltrexone from OTPs was defined as having a Part B claim with Healthcare Common Procedure Coding System (HCPCS) codes corresponding to receipt of these medications (eTable 1 in the Supplement).

To measure MOUD retention among beneficiaries receiving MOUD during the study period, we calculated by cohort the mean and SD and median and IQR for the proportion of eligible days beneficiaries received MOUD. Eligible days were defined as the total number of days from beneficiary date of entry into the cohort through end of the study period. In addition, we calculated the percentage of beneficiaries receiving MOUD on 80% or more of eligible days.^31^ Each measure was stratified by type of MOUD received.

Receipt of any OUD-related and behavioral health-related services was defined as a Medicare Part A or B service claim with an OUD diagnosis or any behavioral health-related code, respectively, on the claim. Telehealth services were defined as a service with any telehealth service code, defined by either Place of Service (POS) code 02 and/or a combination of HCPCS modifier codes and HCPCS or *Current Procedural Terminology* codes included in the CMS list of covered telehealth services noted on the claim (eTable 1 in the Supplement). Experiencing medically treated overdose was based on having a drug overdose (poisoning) *ICD*-*10*-*CM* code on a Medicare Part A or B service claim at any point during the index period through the follow-up period (eTable 1 in the Supplement).

### Demographic and Clinical Covariates

Demographic characteristics included sex, age group, race and ethnicity, census region, county urban-rural status, Medicare eligibility and status, time spent in nursing homes from baseline through follow-up period, co-occurring SUDs, mental health diagnoses, and chronic medical conditions at baseline in the same year as the index visit (eTable 1 in the Supplement). Demographic characteristics were collected by the Social Security Administration and sent to CMS when a beneficiary enrolled in Medicare.

### Statistical Analysis

Characteristics were examined by cohort and reported as frequencies and percentages. We also examined by cohort descriptive statistics for receipt of MOUD and MOUD retention, receipt of any OUD-related and behavioral health-related telehealth services, and experiencing medically treated overdose. To assess differences between cohorts, we used χ^2^ tests for proportions and percentages, *t* tests for means, and Wilcoxon signed rank test for median values.

For the pandemic cohort, 4 multilevel logistic regression models were used to examine characteristics associated with (1) receipt of OUD-related telehealth services; (2) receipt of behavioral health-related telehealth services; (3) MOUD retention (ie, 80% or more of eligible days) among those receiving MOUD; and (4) experiencing medically treated overdose. All models adjusted for baseline demographic and clinical characteristics; beneficiary state of residence was included in models as a level 2 random intercepts parameter to adjust for similarity of same-state beneficiaries. Based on research demonstrating individuals dually eligible for Medicare and Medicaid have increased prevalence of opioid use disorder and elevated risk for overdose,^32,33^ we replicated the 4 multilevel logistic regression analyses stratified by dual status as a sensitivity analysis for our outcomes of interest. Results are presented as adjusted odds ratios (aORs) and corresponding 95% CIs. Multicollinearity was assessed with variance inflation factors and was not identified in final models based on a variance inflation factor cutoff greater than 5. Statistical significance was set at *P* < .05, and all *P* values were 2-tailed. Analyses were conducted with SAS Enterprise Guide version 7.1 (SAS Institute) and Stata version 17.0 (StataCorp).

## Results

### Characteristics of Study Population

The pre–COVID-19 pandemic cohort comprised 105 240 beneficiaries; of these, 61 152 (58.1%) were female, 71 152 (67.6%) were aged 45 to 74 years, and 82 822 (79.5%) non-Hispanic White. The COVID-19 pandemic cohort comprised 70 538 beneficiaries; of these, 40 257 (57.1%) were female, 46 793 (66.3%) were aged 45 to 74 years, and 55 510 (79.7%) were non-Hispanic White (Table 1). The mean (range) observation time was 280 (182-366) days for the prepandemic cohort and 275 (182-365) days for the pandemic cohort. In both cohorts, most beneficiaries were living in metropolitan areas and had both Medicare and Medicaid coverage. Co-occurring SUDs, psychiatric disorders, and other chronic medical conditions were common among both cohorts. A larger percentage of beneficiaries in the pandemic cohort (7084 [10.0%]) died from any cause compared with the prepandemic cohort (8147 [7.7%]; *P* < .001).

**Table 1.** Baseline Demographic and Clinical Characteristics of Medicare Fee-for-Service Beneficiaries With Opioid Use Disorder by Cohort

Characteristic	No. (%)		*P* value^a^
	Pre–COVID-19 pandemic cohort	COVID-19 pandemic cohort	
Cohort size	105 240 (59.87)	70 538 (40.13)	NA
Sex			
Female	61 152 (58.11)	40 257 (57.07)	<.001
Male	44 088 (41.89)	30 281 (42.93)	
Age group, y			
18-44	11 767 (11.18)	7780 (11.03)	<.001
45-64	37 514 (35.65)	22 990 (32.59)	
65-74	33 638 (31.96)	23 803 (33.74)	
≥75	22 321 (21.21)	15 965 (22.63)	
Race and ethnicity			
Non-Hispanic African American	12 393 (11.90)	7868 (11.30)	<.001
Non-Hispanic American Indian or Alaska Native	1079 (1.04)	741 (1.06)	
Non-Hispanic Asian or Pacific Islander	892 (0.86)	694 (1.00)	
Hispanic	6450 (6.19)	4424 (6.36)	
Non-Hispanic White	82 822 (79.52)	55 510 (79.74)	
Other race	510 (0.49)	373 (0.54)	
US Census region			
Northeast	18 487 (17.57)	11 069 (15.69)	<.001
Midwest	19 836 (18.85)	12 729 (18.05)	
South	41 667 (39.60)	29 110 (41.27)	
West	25 231 (23.98)	17 619 (24.98)	
County urban-rural status			
Metropolitan	81 443 (77.39)	55 269 (78.36)	<.001
Micropolitan	21 145 (20.09)	13 603 (19.29)	
Rural	2644 (2.51)	1657 (2.35)	
Dual status			
Medicare only	47 865 (45.48)	33 782 (47.89)	<.001
Medicare and Medicaid	57 375 (54.52)	36 756 (52.11)	
Eligibility			
Aged	55 183 (52.44)	39 031 (55.33)	<.001
Disabled	47 704 (45.33)	29 522 (41.85)	
ESRD	2353 (2.24)	1985 (2.81)	
Other substance use disorder diagnosis			
Alcohol	12 205 (11.60)	8215 (11.65)	.75
Tobacco	38 742 (36.81)	24 181 (34.28)	<.001
Cannabis	7817 (7.43)	5236 (7.42)	.97
Cocaine	4724 (4.49)	2907 (4.12)	<.001
Stimulant	5030 (4.78)	3625 (5.14)	<.001
Sedative/hypnotic	6634 (6.30)	4683 (6.64)	.005
Other psychoactive substance	12 350 (11.74)	7590 (10.76)	<.001
≥2 Substances	20 036 (19.04)	12 944 (18.35)	<.001
Mental health diagnosis			
Anxiety	58 671 (55.75)	39 163 (55.52)	.34
Bipolar disorder	17 454 (16.58)	11 737 (16.64)	.76
Major depression	58 275 (55.37)	38 323 (54.33)	<.001
Personality disorder	6665 (6.33)	4262 (6.04)	.01
Attention-deficit/hyperactivity disorder	5319 (5.05)	3544 (5.02)	.78
Posttraumatic stress disorder	8895 (8.45)	5953 (8.44)	.93
Schizophrenia or other psychotic disorder	9849 (9.36)	6934 (9.83)	.001
≥2 Mental health diagnoses	50 026 (47.54)	33 177 (47.03)	.04
Other chronic medical conditions			
Cancer	11 347 (10.78)	7921 (11.23)	.003
Diabetes	38 081 (36.18)	25 555 (36.23)	.85
Cardiovascular and other circulatory	81 124 (77.08)	54 177 (76.81)	.17
Chronic respiratory disease	40 351 (38.34)	25 769 (36.53)	<.001
Viral hepatitis	9098 (8.65)	5457 (7.74)	<.001
HIV	1437 (1.37)	965 (1.37)	.96
Obesity	38 749 (36.82)	25 537 (36.20)	.009
Liver disease cirrhosis and other liver conditions^b^	14 132 (13.43)	9725 (13.79)	.03
Acute/chronic pain	93 616 (88.95)	61 912 (87.77)	<.001

Abbreviations: ESRD, end-stage renal disease; NA, not applicable.

^a^ To assess differences between cohorts, we used χ^2^ tests for proportions and percentages.

^b^ Excluding hepatitis.

### Receipt of Telehealth Services

A larger percentage of beneficiaries in the pandemic cohort compared with the prepandemic cohort received any telehealth service (48 390 [68.6%] vs 2594 [2.5%]; *P* < .001), behavioral health-related telehealth services (28 902 [41.0%] vs 1967 [1.9%]; *P* < .001), and OUD-related telehealth services (13 829 [19.6%] vs 593 [0.6%]; *P* < .001) (Table 2). At the baseline visit, 12.1% (8516 of 70 538) in the pandemic cohort received an OUD-related telehealth service vs 0.1% (125 of 105 240) in the prepandemic cohort (*P* < .001).

**Table 2.** Telehealth, Medications for Opioid Use Disorder (MOUD) Receipt and Retention, and Medically Treated Overdose Among Medicare Fee-for-Service Beneficiaries With Opioid Use Disorder (OUD)

Measure	No. (%)		*P* value^a^
	Pre–COVID-19 pandemic cohort (n = 105 240)	COVID-19 pandemic cohort (n = 70 538)	
**Receipt of telehealth services**			
Receipt of telehealth services at baseline visit			
Any telehealth service	212 (0.20)	9782 (13.87)	<.001
Behavioral health-related telehealth service	179 (0.17)	8704 (12.34)	<.001
OUD-related telehealth service	125 (0.12)	8516 (12.07)	<.001
Receipt of telehealth services from baseline visit through follow-up period			
Any telehealth service	2594 (2.46)	48 390 (68.60)	<.001
Behavioral health-related telehealth service	1967 (1.87)	28 902 (40.97)	<.001
OUD-related telehealth service	593 (0.56)	13 829 (19.61)	<.001
**Receipt of MOUD**			
Receipt of MOUD at baseline visit			
Any MOUD from OTP or pharmacy	4667 (4.43)	5321 (7.54)	<.001
Any MOUD from OTP	0	2087 (2.96)	<.001
Buprenorphine from OTP	0	87 (0.12)	<.001
ER naltrexone from OTP	0	0	
Methadone from OTP	0	2002 (2.84)	<.001
Any MOUD from pharmacy	4667 (4.43)	3242 (4.60)	.11
Buprenorphine from pharmacy	4566 (4.34)	3184 (4.51)	.08
ER naltrexone from pharmacy	102 (0.10)	58 (0.08)	.32
Receipt of MOUD from baseline visit through follow-up period			
Any MOUD from OTP or pharmacy	11 360 (10.79)	8854 (12.55)	<.001
Any MOUD from OTP	1451 (1.38)	2837 (4.02)	<.001
Buprenorphine from OTP	44 (0.04)	161 (0.23)	<.001
ER naltrexone from OTP	0	1 (0.00)	.22
Methadone from OTP	1419 (1.35)	2719 (3.85)	<.001
Any MOUD from pharmacy	9990 (9.49)	6174 (8.75)	<.001
Buprenorphine from pharmacy	9675 (9.19)	6005 (8.51)	<.001
ER naltrexone from pharmacy	369 (0.35)	198 (0.28)	.01
**MOUD retention among those receiving MOUD**			
Buprenorphine from pharmacies only			
Proportion of eligible days covered, mean (SD)	0.51 (0.34)	0.52 (0.34)	.13
Proportion of eligible days covered, median (IQR)	0.55 (0.15-0.84)	0.56 (0.15-0.86)	.03
Beneficiaries with ≥80% adherence, %	31.07	33.26	.005
ER naltrexone from pharmacies only			
Proportion of eligible days covered, mean (SD)	0.35 (0.26)	0.37 (0.28)	.44
Proportion of eligible days covered, median (IQR)	0.29 (0.12-0.51)	0.30 (0.12-0.56)	.60
Beneficiaries with ≥80% adherence, %	7.94	11.83	.16
**MOUD from OTPs only**			
Proportion of eligible days covered			
Mean (SD)	0.16 (0.06)	0.69 (0.30)	<.001
Median (IQR)	0.16 (0.13-0.20)	0.81 (0.46-0.96)	<.001
Beneficiaries with ≥80% adherence, %	0.00	50.93	<.001
**MOUD from OTPs and pharmacies**			
Proportion of eligible days covered			
Mean (SD)	0.29 (0.17)	0.58 (0.27)	<.001
Median (IQR)	0.25 (0.17-0.38)	0.65 (0.38-0.80)	<.001
Beneficiaries with ≥80% adherence, %	2.47	26.11	<.001
Medically treated overdose from index period through follow-up period			
Any medically treated overdose	19 491 (18.52)	13 004 (18.44)	.65

Abbreviations: ER, extended-release; OTP, opioid treatment program.

^a^ To assess differences between cohorts, we used χ^2^ tests for proportions and percentages, *t* tests for means, and the Wilcoxon signed rank test for medians.

### Receipt of MOUD

At the baseline visit, few beneficiaries with OUD in both cohorts received any MOUD, with a larger percentage of the pandemic cohort receiving any MOUD (5321 [7.5%] vs 4667 [4.4%]; *P* < .001) (Table 2). This was because of a larger percentage of the pandemic cohort receiving MOUD from an OTP, primarily methadone (2002 [2.8%] vs 0; *P* < .001). At the index visit, there were no differences between cohorts for receiving buprenorphine (3184 [4.5%] vs 4566 [4.3%]; *P* = .08) or ER naltrexone (58 [0.1%] vs 102 [0.1%]; *P* = .32) from pharmacies.

During the study period, a larger percentage of the pandemic cohort received any MOUD (8854 [12.6%] vs 11 360 [10.8%]; *P* < .001). Receipt of MOUD from an OTP, primarily methadone, was more common in the pandemic cohort (2837 [4.0%] vs 1451 [1.4%]; *P* < .001), whereas receiving buprenorphine (9675 [9.2%] vs 6005 [8.5%]; *P* < .001) and ER naltrexone (369 [0.4%] vs 198 [0.3%]; *P* = .01) from pharmacies was more common in the prepandemic cohort.

### MOUD Retention

Among beneficiaries receiving MOUD, there was no difference between cohorts for mean proportion of eligible days receiving buprenorphine; however, median eligible days and the percentage of beneficiaries receiving buprenorphine on 80% or more of eligible days were higher in the pandemic cohort (Table 2). There was no difference between cohorts for mean or median proportions of eligible days receiving ER naltrexone, nor for percentage receiving ER naltrexone on 80% or more of eligible days. The mean and median proportions of eligible days beneficiaries received MOUD from OTPs and the percentage receiving MOUD from OTPs on 80% or more of eligible days were higher in the pandemic cohort, likely reflecting the initial implementation of the OTP payment policy only in the last 2 months of the prepandemic cohort follow-up period.

### Experiencing Medically Treated Overdose

During the study period, the percentage of beneficiaries experiencing a medically treated overdose was similar between cohorts, including 18.5% (19 491 of 105 240) in the prepandemic cohort and 18.4% (13 004 of 70 538) in the pandemic cohort (*P* = .65) (Table 2).

### Characteristics Associated With Receipt of OUD and Behavioral Health-Related Telehealth Services in the Pandemic Cohort

Among the pandemic cohort, receipt of OUD-related telehealth services was more likely among those receiving MOUD from OTPs and pharmacies (aOR, 3.30; 95% CI, 2.39-4.57), ER naltrexone from pharmacies only (aOR, 1.49; 95% CI, 1.04-2.14), or buprenorphine from pharmacies only (aOR, 3.60; 95% CI, 3.39-3.82) compared with not receiving MOUD; receipt of MOUD from OTPs only was associated with lower odds of receiving OUD-related telehealth services (aOR, 0.35; 95% CI, 0.30-0.40) (Table 3). Multiple demographic characteristics, including race and ethnicity and sex, as well as clinical characteristics were associated with receipt of OUD-related telehealth services. Results for receipt of behavioral health-related telehealth services were generally consistent with those for OUD-related telehealth services.

**Table 3.** Characteristics Associated With Receipt of Opioid Use Disorder (OUD)–Related Telehealth Service and Behavioral Health-Related Telehealth Services During Study Period Among Beneficiaries With OUD in the Pandemic Cohort^a^

Characteristic	aOR (95% CI)^b^	
	OUD-related telehealth services	Behavioral health-related telehealth services
Total, No.	70 497	70 497
Receipt of MOUD during study period		
No MOUD	1 [Reference]	1 [Reference]
MOUD from OTP only	0.346 (0.299-0.401)^c^	0.664 (0.603-0.730)^c^
MOUD from OTP and pharmacy	3.302 (2.385-4.573)^c^	1.333 (0.944-1.880)
ER naltrexone from pharmacy only	1.491 (1.039-2.138)^c^	1.542 (1.083-2.195)^c^
Buprenorphine from pharmacy only	3.599 (3.389-3.821)^c^	2.007 (1.889-2.134)^c^
Baseline sex		
Female	1.099 (1.054-1.145)^c^	1.223 (1.181-1.267)^c^
Male	1 [Reference]	1 [Reference]
Baseline age group, y		
18-44	1 [Reference]	1 [Reference]
45-64	0.942 (0.878-1.010)	1.013 (0.953-1.077)
65-74	0.927 (0.856-1.003)	0.882 (0.824-0.944)^c^
≥75	0.917 (0.840-1.003)	0.754 (0.699-0.814)^c^
Baseline race and ethnicity		
Non-Hispanic African American	0.919 (0.857-0.986)^c^	0.906 (0.856-0.960)^c^
Non-Hispanic American Indian or Alaska Native	1.066 (0.878-1.295)	1.013 (0.859-1.194)
Non-Hispanic Asian or Pacific Islander	1.211 (1.008-1.454)^c^	1.147 (0.973-1.353)
Hispanic	0.994 (0.916-1.078)	0.968 (0.902-1.038)
Non-Hispanic White	1 [Reference]	1 [Reference]
Other race	1.018 (0.785-1.319)	1.034 (0.828-1.291)
Baseline US census region		
Northeast	1 [Reference]	1 [Reference]
Midwest	0.793 (0.591-1.064)	0.701 (0.552-0.889)^c^
South	0.652 (0.497-0.856)^c^	0.632 (0.508-0.788)^c^
West	0.828 (0.620-1.105)	0.783 (0.620-0.989)^c^
Baseline county urban-rural status		
Metropolitan	1 [Reference]	1 [Reference]
Micropolitan	0.965 (0.915-1.018)	0.880 (0.842-0.920)^c^
Rural	0.955 (0.832-1.097)	0.793 (0.707-0.889)^c^
Baseline dual status		
Medicare only	1 [Reference]	1 [Reference]
Medicare and Medicaid	1.005 (0.957-1.054)	1.041 (1.000-1.084)^c^
Time spent in nursing home from baseline to end of study, quartile		
None	1 [Reference]	1 [Reference]
1st	0.609 (0.532-0.697)^c^	0.655 (0.594-0.723)^c^
2nd	0.674 (0.591-0.768)^c^	0.824 (0.748-0.908)^c^
3rd	0.600 (0.525-0.686)^c^	0.842 (0.766-0.924)^c^
4th	0.647 (0.569-0.736)^c^	0.870 (0.793-0.954)^c^
Baseline other substance use disorder diagnosis^d^		
Alcohol	0.937 (0.875-1.003)	1.097 (1.038-1.161)^c^
Tobacco	0.891 (0.850-0.934)^c^	1.071 (1.030-1.114)^c^
Cannabis	0.872 (0.802-0.949)^c^	0.957 (0.893-1.027)
Cocaine	0.953 (0.852-1.066)	0.862 (0.786-0.946)^c^
Stimulant	0.885 (0.799-0.980)^c^	0.757 (0.695-0.824)^c^
Sedative/hypnotic	1.563 (1.453-1.681)^c^	1.432 (1.340-1.530)^c^
Other psychoactive substance	0.776 (0.721-0.835)^c^	0.923 (0.869-0.980)^c^
Baseline mental health diagnosis^d^		
Anxiety	0.983 (0.939-1.028)	1.576 (1.518-1.637)^c^
Bipolar disorder	1.077 (1.015-1.143)^c^	1.664 (1.585-1.748)^c^
Major depression	1.123 (1.075-1.174)^c^	1.986 (1.915-2.060)^c^
Personality disorder	0.946 (0.868-1.031)	1.201 (1.117-1.291)^c^
Attention-deficit/hyperactivity disorder	1.047 (0.957-1.146)	1.429 (1.320-1.548)^c^
Posttraumatic stress disorder	1.165 (1.082-1.254)^c^	1.572 (1.472-1.679)^c^
Schizophrenia or other psychotic disorder	0.840 (0.778-0.907)^c^	1.384 (1.301-1.472)^c^
Baseline other chronic medical conditions^d^		
Cancer	0.887 (0.831-0.946)^c^	0.913 (0.866-0.962)^c^
Diabetes	0.928 (0.888-0.970)^c^	0.958 (0.923-0.994)^c^
Cardiovascular and other circulatory	0.835 (0.793-0.878)^c^	0.889 (0.850-0.929)^c^
Chronic respiratory disease	0.866 (0.829-0.905)^c^	0.964 (0.929-0.999)^c^
Viral hepatitis	1.113 (1.029-1.204)^c^	0.924 (0.865-0.986)^c^
HIV	1.144 (0.967-1.353)	1.192 (1.035-1.373)^c^
Obesity	1.033 (0.989-1.079)	1.057 (1.019-1.096)^c^
Liver disease cirrhosis and other liver conditions^e^	0.792 (0.744-0.843)^c^	0.878 (0.835-0.922)^c^
Acute/chronic pain	1.062 (0.995-1.113)	1.105 (1.045-1.169)^c^

Abbreviations: aOR, adjusted odd ratio; ER, extended-release; MOUD, medications for opioid use disorder; OTP, opioid treatment program.

^a^ To enable mutually exclusive groups for the regression analysis, 27 beneficiaries (0.3% of beneficiaries receiving MOUD) that received both buprenorphine and ER naltrexone from pharmacies during the study period were excluded from the analysis.

^b^ Models adjusted for all variables in the table.

^c^ *P* < .05.

^d^ Reference group is not having condition.

^e^ Excluding hepatitis.

### Characteristics Associated With MOUD Retention in the Pandemic Cohort

Among the 8854 beneficiaries (12.6%) in the pandemic cohort receiving MOUD during the study period, receipt of OUD-related telehealth services was associated with increased odds of receiving MOUD on 80% or more of eligible days (aOR, 1.27; 95% CI, 1.14-1.41). Lower odds were found among those receiving MOUD from both OTPs and pharmacies (aOR, 0.39; 95% CI, 0.26-0.56), ER naltrexone from pharmacies only (aOR, 0.22; 95% CI, 0.13-0.36), or buprenorphine from pharmacies only (aOR, 0.57; 95% CI, 0.51-0.64) compared with MOUD from an OTP only. Multiple demographic characteristics, including race and ethnicity, and clinical characteristics were associated with MOUD retention (Table 4).

**Table 4.** Characteristics Associated With Medications for Opioid Use Disorder (MOUD) Retention for at Least 80% of Eligible Days During the Study Period Among Beneficiaries With Opioid Use Disorder (OUD) in the Pandemic Cohort Receiving MOUD During the Study Period^a^

Characteristic	aOR (95% CI)^b^
Total, No.	8826
Receipt of OUD-related telehealth service	1.267 (1.139-1.410)^c^
Receipt of MOUD during study period	
MOUD from OTP only	1 [Reference]
MOUD from OTP and pharmacy	0.385 (0.264-0.563)^c^
ER naltrexone from pharmacy only	0.217 (0.132-0.357)^c^
Buprenorphine from pharmacy only	0.572 (0.509-0.644)^c^
Baseline sex	
Female	0.910 (0.827-1.002)
Male	1 [Reference]
Baseline age group, y	
18-44	1 [Reference]
45-64	1.226 (1.085-1.386)^c^
65-74	1.125 (0.963-1.315)
≥75	0.820 (0.635-1.058)
Baseline race and ethnicity	
Non-Hispanic African American	0.699 (0.592-0.827)^c^
Non-Hispanic American Indian or Alaska Native	0.901 (0.587-1.381)
Non-Hispanic Asian or Pacific Islander	0.973 (0.563-1.683)
Hispanic	0.845 (0.694-1.027)
Non-Hispanic White	1 [Reference]
Other race	0.660 (0.311-1.400)
Baseline US census region	
Northeast	1 [Reference]
Midwest	0.994 (0.754-1.310)
South	1.015 (0.800-1.289)
West	0.761 (0.581-0.997)^c^
Baseline county urban-rural status	
Metropolitan	1 [Reference]
Micropolitan	0.943 (0.834-1.067)
Rural	1.000 (0.727-1.374)
Baseline dual status	
Medicare only	1 [Reference]
Medicare and Medicaid	1.204 (1.070-1.354)^c^
Time spent in nursing home from baseline to end of study, quartile	
None	1 [Reference]
1st	0.338 (0.193-0.594)^c^
2nd	0.583 (0.357-0.951)^c^
3rd	0.024 (0.003-0.177)^c^
4th	0.272 (0.105-0.702)^c^
Baseline other substance use disorder diagnosis^d^	
Alcohol	0.745 (0.637-0.871)^c^
Tobacco	0.950 (0.859-1.050)
Cannabis	0.753 (0.626-0.906)^c^
Cocaine	0.560 (0.439-0.712)^c^
Stimulant	0.546 (0.444-0.672)^c^
Sedative/hypnotic	0.872 (0.709-1.072)
Other psychoactive substance	0.983 (0.859-1.124)
Baseline mental health diagnosis^d^	
Anxiety	0.913 (0.818-1.019)
Bipolar disorder	1.027 (0.903-1.168)
Major depression	0.905 (0.814-1.006)
Personality disorder	0.717 (0.579-0.888)^c^
Attention-deficit/hyperactivity disorder	0.926 (0.767-1.117)
Posttraumatic stress disorder	1.037 (0.884-1.217)
Schizophrenia or other psychotic disorder	0.782 (0.657-0.929)^c^
Baseline other chronic medical conditions^d^	
Cancer	0.709 (0.565-0.890)^c^
Diabetes	0.921 (0.817-1.038)
Cardiovascular and other circulatory	0.888 (0.795-0.992)^c^
Chronic respiratory disease	0.872 (0.778-0.977)^c^
Viral hepatitis	0.900 (0.781-1.037)
HIV	0.731 (0.514-1.039)
Obesity	1.125 (1.004-1.261)^c^
Liver disease cirrhosis and other liver conditions^e^	0.829 (0.706-0.973)^c^
Acute/chronic pain	0.838 (0.750-0.936)^c^

Abbreviations: aOR, adjusted odd ratio; ER, extended-release; OTPs, opioid treatment programs.

^a^ To enable mutually exclusive groups for the regression analysis, 27 beneficiaries (0.3% of beneficiaries receiving MOUD) that received both buprenorphine and ER naltrexone from pharmacies during the study period were excluded from the analysis.

^b^ Models adjusted for all variables in the table.

^c^ *P* < .05.

^d^ Reference group is not having condition.

^e^ Excluding hepatitis.

### Characteristics Associated With Experiencing Medically Treated Overdose in the Pandemic Cohort

Approximately 18% (13 044 of 70 538) of the pandemic cohort experienced medically treated overdose during the study period. Lower adjusted odds of experiencing medically treated overdose were seen among beneficiaries receiving OUD-related telehealth services (aOR, 0.67; 95% CI, 0.63-0.71) and those receiving MOUD from OTPs only (aOR, 0.54; 95% CI, 0.47-0.63) or buprenorphine from pharmacies only (aOR, 0.91; 95% CI, 0.84-0.98) compared with receiving no MOUD (Table 5). Demographic characteristics, including race and ethnicity, as well as clinical characteristics were also associated with experiencing medically treated overdose.

**Table 5.** Characteristics Associated With Experiencing Any Medically Attended Overdose During Study Period Among Beneficiaries With Opioid Use Disorder (OUD) in the Pandemic Cohort^a^

Characteristic	aOR (95% CI)^b^
Total, No.	70 497
Receipt of OUD-related telehealth service	0.671 (0.634-0.710)^c^
Receipt of MOUD during study period	
No MOUD	1 [Reference]
MOUD from OTP only	0.540 (0.465-0.628)
MOUD from OTP and pharmacy	1.367 (0.920-2.032)
ER naltrexone from pharmacy only	0.889 (0.613-1.288)
Buprenorphine from pharmacy only	0.905 (0.836-0.980)
Baseline sex	
Female	1.072 (1.026-1.119)
Male	1 [Reference]
Baseline age group, y	
18-44	1 [Reference]
45-64	0.699 (0.650-0.753)
65-74	0.701 (0.645-0.761)
≥75	0.650 (0.593-0.714)
Baseline race and ethnicity	
Non-Hispanic African American	1.082 (1.011-1.159)
Non-Hispanic American Indian or Alaska Native	1.029 (0.844-1.253)
Non-Hispanic Asian or Pacific Islander	1.233 (1.007-1.509)
Hispanic	0.917 (0.839-1.004)
Non-Hispanic White	1 [Reference]
Other race	1.333 (1.029-1.727)
Baseline US Census region	
Northeast	1 [Reference]
Midwest	0.996 (0.871-1.139)
South	0.870 (0.770-0.983)
West	1.026 (0.897-1.173)
Baseline county urban-rural status	
Metropolitan	1 [Reference]
Micropolitan	0.912 (0.863-0.963)
Rural	1.033 (0.901-1.185)
Baseline dual status	
Medicare only	1 [Reference]
Medicare and Medicaid	0.856 (0.814-0.900)
Time spent in nursing home from to end of study, quartile	
None	1 [Reference]
1st	1.982 (1.799-2.184)
2nd	1.689 (1.527-1.869)
3rd	1.855 (1.684-2.044)
4th	1.116 (1.003-1.243)
Baseline other substance use disorder diagnosis^d^	
Alcohol	1.179 (1.108-1.255)
Tobacco	1.187 (1.132-1.245)
Cannabis	1.233 (1.144-1.330)
Cocaine	1.435 (1.305-1.578)
Stimulant	1.302 (1.190-1.425)
Sedative/hypnotic	1.313 (1.220-1.413)
Other psychoactive substance	1.787 (1.675-1.905)
Baseline mental health diagnosis^d^	
Anxiety	1.387 (1.321-1.456)
Bipolar disorder	1.143 (1.079-1.211)
Major depression	1.301 (1.241-1.364)
Personality disorder	1.126 (1.041-1.219)
Attention-deficit/hyperactivity disorder	1.010 (0.924-1.104)
Posttraumatic stress disorder	0.916 (0.852-0.986)
Schizophrenia or other psychotic disorder	1.142 (1.067-1.222)
Baseline other chronic medical conditions^d^	
Cancer	1.902 (1.796-2.013)
Diabetes	1.156 (1.106-1.209)
Cardiovascular and other circulatory	1.461 (1.375-1.553)
Chronic respiratory disease	1.350 (1.293-1.410)
Viral hepatitis	1.050 (0.975-1.130)
HIV	0.971 (0.821-1.148)
Obesity	1.060 (1.014-1.107)
Liver disease cirrhosis and other liver conditions^e^	1.569 (1.487-1.656)
Acute/chronic pain	1.308 (1.214-1.410)

Abbreviations: aOR, adjusted odd ratio; ER, extended-release; MOUD, medications for opioid use disorder; OTPs, opioid treatment programs.

^a^ To enable mutually exclusive groups for the regression analysis, 27 beneficiaries (0.3% of beneficiaries receiving MOUD) that received both buprenorphine and ER naltrexone from pharmacies during the study period were excluded from the analysis.

^b^ Models adjusted for all variables in the table.

^c^ *P* < .05.

^d^ Reference group is not having condition.

^e^ Excluding hepatitis.

### Sensitivity Analyses

Results from the multilevel logistic regression analyses examining characteristics associated with receipt of OUD (eTable 2 in the Supplement), behavioral health-related telehealth services (eTable 3 in the Supplement), MOUD retention (eTable 4 in the Supplement), and experiencing medically treated overdose (eTable 5 in the Supplement) stratified by dual eligibility status (ie, Medicare and Medicaid as well as Medicare only) were generally consistent with findings from the primary analyses.

## Discussion

In this national study of Medicare beneficiaries from September 2018 to February 2021, only a small proportion received MOUD following an index OUD diagnosis, with a slightly larger proportion in the pandemic cohort receiving MOUD during the study period. Consistent with the broadening of telehealth use during the pandemic, approximately 1 in 8 beneficiaries in the pandemic cohort received OUD-related telehealth services at their index OUD visit compared with 1 in 800 in the prepandemic cohort. Further, the percentage of the pandemic cohort receiving OUD-related telehealth services was 35-fold that of the prepandemic cohort, documenting marked increases in telehealth use for OUD during the pandemic.

Given interest in understanding if emergency authorities related to OUD treatment would result in differences in patient outcomes, our results are particularly valuable. Among those receiving MOUD, treatment retention in the pandemic cohort was either no different than or higher than retention in the prepandemic cohort, depending on type of MOUD received. Additionally, the percentage of each cohort experiencing a medically treated overdose during the study period was similar, an encouraging finding given the 30% increase in overdose deaths in the US between 2019 and 2020.^3^ Importantly, among the pandemic cohort, after adjusting for MOUD receipt, demographic background, and clinical characteristics, beneficiaries who received OUD-related telehealth services had lower odds of experiencing medically treated overdose, and among the subset of beneficiaries receiving MOUD, those receiving OUD-related telehealth services had elevated odds for improved MOUD retention. These findings are consistent with a 2022 systematic review,^34^ which found that among the small number of studies conducted prior to the pandemic, telehealth was found to be as effective as in-person treatment for SUD in terms of retention, therapeutic alliance, and substance use.

Despite these promising findings, in both cohorts, only a small proportion of individuals receiving MOUD received medications on 80% or more of eligible days. This finding underscores the ongoing challenges in engaging and retaining patients in treatment. Research suggests potential for contingency management to improve retention in some patients receiving MOUD.^35^ Digital application–supported treatment has also been suggested as a useful approach to enhance MOUD adherence.^36^ Implementation of these or other innovative approaches are needed to improve retention in care and patient recovery outcomes.

A novel finding in this study relates to implementation of the Medicare OTP episode of care payment policy in January 2020. This policy appears to have provided a new avenue for treatment for both cohorts. MOUD from OTPs was absent at baseline in the prepandemic cohort, increasing to 1.4% by the end of the follow-up period, which included the first 2 months after this coverage went into effect; in the pandemic cohort, MOUD from OTPs was even more common—observed in 3.0% at baseline and 4.0% by the end of follow-up. In addition, retention by type of MOUD was strongest among those receiving MOUD from OTPs during the pandemic cohort, a time when new emergency flexibilities to provide additional take-home doses of methadone were implemented. This change may have contributed to the higher rates of retention seen in the pandemic cohort. Future research should further examine the outcomes of these policy changes.

Our results highlight the continued need for a well-coordinated and comprehensive health system that integrates physical and behavioral health care. Beneficiaries in both cohorts had many challenging health conditions—co-occurring SUDs, mental health diagnoses, and chronic medical conditions, including a large majority with chronic pain. Our observations align with the goals specified in the CMS Behavioral Health Strategy and identify opportunities for focused and coordinated actions across clinical settings and health care practitioners,^37^ including advancing integrated care for co-occurring physical and behavioral health conditions, which is a core component of the 2021 HHS overdose prevention strategy.^38^

Health inequities were consistent with previous research showing less access to and provision of MOUD for certain racial and ethnic minority persons.^39,40^ In particular, we found non-Hispanic African American persons had lower odds of receiving OUD or behavioral health-related telehealth services and lower odds for MOUD retention. In addition, we found higher odds of medically treated overdose among persons who were non-Hispanic African American, American Indian or Alaska Native, and Asian or Pacific Islander. Tailored and culturally appropriate policy and programmatic interventions are needed to address disparities in accessing OUD treatment, overdose prevention, and recovery support services given recent sharp increases in overdose deaths among these groups.^38,41,42^

While this study addressed use of telehealth services by persons with OUD and included MOUD retention as a measure of treatment quality, future research should examine the outcomes of telehealth services on other health outcomes, such as continued illicit opioid use and overdose deaths. In addition, research is needed to understand barriers and facilitators of clinician and patient use of telehealth. Ultimately, approaches are needed that facilitate making telehealth more person-centered, eliminate the digital divide, and determine whether our findings generalize to other SUDs, pain management, and psychiatric disorders.

### Limitations

This study has limitations. Our study contains Medicare fee-for-service beneficiaries, including a large percentage with disability; thus, OUD prevalence may be higher in this cohort and findings might not generalize to other populations. Our study required individuals to have an *ICD*-*10*-*CM* OUD diagnosis code for inclusion in the study. Although a broad range of *ICD*-*10*-*CM* opioid-related service codes were used to identify the cohort (eTable 1 in the Supplement), it is possible that some individuals with OUD were not ascertained as having an OUD diagnosis and are thus not included in this study. Consistent with studies using claims data, we assumed medications were used as dispensed.^43^ Provision of telehealth may be underestimated, as differential uptake by clinicians and health systems of telehealth billing codes and modifiers added during initial phases of the pandemic may have occurred. OTPs were not required by CMS to report telehealth modifier codes; thus, OTP telehealth services may be underestimated. Claims data lack information that might be associated with MOUD retention, such as severity of OUD. Despite using parallel time frames to construct each cohort (September 2018 to February 2020 and September 2019 to February 2021), the size of the pandemic cohort was smaller than the prepandemic cohort. This difference appears to be consistent with the general pattern of fewer Medicare beneficiaries seeking services during the pandemic compared with prepandemic levels^44^; however, it may have introduced selection bias for individuals entering the pandemic cohort and influenced examined outcomes. There was also a larger percentage of deaths in the pandemic cohort compared with the prepandemic cohort, and this may have influenced findings; however, the underlying cause of these deaths is not available at this time. Future research should examine the underlying causes of death among Medicare beneficiaries with OUD to improve understanding of these patterns. Although many of the findings are statistically significant, the absolute differences are relatively small; the clinical significance of these difference may not be substantially different. Additionally, given the observational nature of the study, we cannot draw causal inferences for our findings. Despite these limitations, to our knowledge, this study is the first to examine and compare the use of OUD and behavioral health-related telehealth services, receipt and retention of MOUD, and experiencing medically treated overdose using a large national data set.

## Conclusions

Authorities to facilitate use of telehealth and provision of MOUD during the COVID-19 pandemic were used by Medicare beneficiaries initiating new episodes of OUD-related care. Use of telehealth during the pandemic was associated with improved retention in care and reduced odds of medically treated overdose, providing support for permanent adoption. Strategies to expand provision of MOUD, increase retention in care, and address co-occurring physical and behavioral health conditions are urgently needed in the context of an escalating overdose crisis.

## Notes

Supplement. **eFigure.** Cohort Preindex, Index, and Follow-up Periods for the Prepandemic and Pandemic Cohorts **eTable 1.** Definitions and Billing Codes Used for OUD, MOUD, Telehealth, Medically Treated Overdose, and Other Conditions and Services Received **eTable 2.** Characteristics Associated with Receipt of OUD-Related Telehealth Services During Study Period Among Beneficiaries With OUD in the Pandemic Cohort by Dual Eligible Status **eTable 3.** Characteristics Associated With Receipt of Behavioral Health-Related Telehealth Services During Study Period Among Beneficiaries With OUD in the Pandemic Cohort by Dual Eligible Status **eTable 4.** Characteristics Associated With MOUD Retention for at Least 80% of Eligible Days During Study Period Among Beneficiaries With OUD in the Pandemic Cohort Receiving MOUD During the Study Period by Dual Eligible Status **eTable 5.** Characteristics Associated With Experiencing Any Medically Treated Drug Overdose During Study Period Among Beneficiaries With OUD in the Pandemic Cohort by Dual Eligible Status
