# Supplementary material for: Receipt of Telehealth Services, Receipt and Retention of Medications for Opioid Use Disorder, and Medically Treated Overdose Among Medicare Beneficiaries Before and During the COVID-19 Pandemic
Source: JAMA Psychiatry. 2022 Aug 31;79(10):981–92. doi: 10.1001/jamapsychiatry.2022.2284 (PMC9434479; doi:10.1001/jamapsychiatry.2022.2284)
Supplement: Supplement. — eFigure. Cohort Preindex, Index, and Follow-up Periods for the Prepandemic and Pandemic Cohorts eTable 1. Definitions and Billing Codes Used for OUD, MOUD, Telehealth, Medically Treated Overdose, and Other Conditions and Services Received eTable 2. Characteristics Associated with Receipt of OUD-Related Telehealth Services During Study Period Among Beneficiaries With OUD in the Pandemic Cohort by Dual Eligible Status eTable 3. Characteristics Associated With Receipt of Behavioral Health-Related Telehealth Services During Study Period Among Beneficiaries With OUD in the Pandemic Cohort by Dual Eligible Status eTable 4. Characteristics Associated With MOUD Retention for at Least 80% of Eligible Days During Study Period Among Beneficiaries With OUD in the Pandemic Cohort Receiving MOUD During the Study Period by Dual Eligible Status eTable 5. Characteristics Associated With Experiencing Any Medically Treated Drug Overdose During Study Period Among Beneficiaries With OUD in the Pandemic Cohort by Dual Eligible Status [file jamapsychiatry-e222284-s001.pdf]

Supplemental Online Content

Jones CM, Shoff C, Hodges K, et al. Receipt of telehealth services, receipt and retention of medications for opioid use disorder, and medically treated overdose among Medicare beneficiaries before and during the COVID-19 pandemic. *JAMA Psychiatry*. Published online August 31, 2022. doi:10.1001/jamapsychiatry.2022.2284

eFigure. Cohort Preindex, Index, and Follow-up Periods for the Prepandemic and Pandemic Cohorts

eTable 1. Definitions and Billing Codes Used for OUD, MOUD, Telehealth, Medically Treated Overdose, and Other Conditions and Services Received

eTable 2. Characteristics Associated with Receipt of OUD-Related Telehealth Services During Study Period Among Beneficiaries With OUD in the Pandemic Cohort by Dual Eligible Status

eTable 3. Characteristics Associated With Receipt of Behavioral Health-Related Telehealth Services During Study Period Among Beneficiaries With OUD in the Pandemic Cohort by Dual Eligible Status

eTable 4. Characteristics Associated With MOUD Retention for at Least 80% of Eligible Days During Study Period Among Beneficiaries With OUD in the Pandemic Cohort Receiving MOUD During the Study Period by Dual Eligible Status

eTable 5. Characteristics Associated With Experiencing Any Medically Treated Drug Overdose During Study Period Among Beneficiaries With OUD in the Pandemic Cohort by Dual Eligible Status

This supplementary material has been provided by the authors to give readers additional information about their work.

© 2022 Jones CM et al. *JAMA Psychiatry*.

eFigure. Cohort Preindex, Index, and Follow-up Periods for the Prepandemic and Pandemic Cohorts

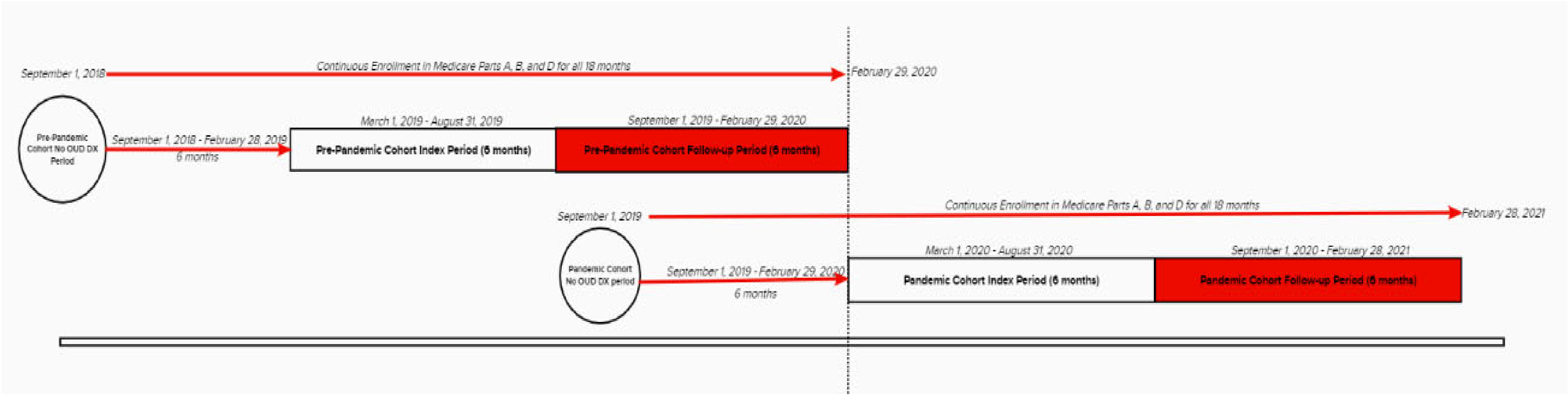

© 2022 Jones CM et al. *JAMA Psychiatry*.

eTable 1. Definitions and Billing Codes Used for OUD, MOUD, Telehealth, Medically Treated Overdose, and Other Conditions and Services Received

Variables	Definition
**Time Spent in Nursing Home**	We considered an individual to be a current nursing home resident on any given day if the most recent assessment was not a discharge assessment and less than 150 days old. An individual without an assessment for 150 days was assumed to be discharged from the facility.
**Medication Received**	**NDCs**
Buprenorphine from pharmacies	59385001201, 59385001230, 59385001401, 59385001430, 59385001601, 59385001630, 00093365640, 00093365740, 00093365840, 00093365940, 42858035340, 42858049340, 42858050103, 42858050203, 42858058640, 42858075040, 42858083940, 55700057904, 58118315608, 60687048121, 60687049221, 62756045964, 62756045983, 62756046064, 62756046083, 67046099430, 67046099530, 70518071100, 70518071101, 70518071102, 70518155700, 70518162500, 70518221700, 70518221800, 71335035301, 71335035302, 71335035303, 71335035304, 71335035305, 71335035306, 71335035307, 71335095001, 71335095002, 71335095003, 71335095004, 71335095005, 71335095006, 71335095007, 71335115401, 71335115402, 71335115403, 71335115404, 71335115405, 71335115406, 71335115407, 71335115408, 71335115409, 55700056804, 00093360040, 00093360140, 00093360240, 00093360340, 67046099230, 67046099330, 60846097003, 60846097103, 63629727001, 63629727002, 67046099830, 67046099930, 00054017613, 00054017713, 00074201201, 00093537856, 00093537956, 00143924605, 00228315303, 00228315603, 00378092393, 00378092493, 00409201232, 21695051510, 35356055530, 35356055630, 40042001001, 42023017901, 42023017905, 43063066706, 50383092493, 50383093093, 54569657800, 55390010010, 55700030230, 55700030330, 58118017608, 58118017708, 63629712601, 63629712602, 63629712603, 63629712604, 63629712605, 63629712606, 63629712607, 63629712608, 63629712609, 68258299103, 68308020230, 68308020830, 70518044200, 70518201400, 70518221600, 70518222600, 71335116301, 71335116302, 71335116303, 71335116304, 71335116305, 71335116306, 71335116307, 71335116308, 71335116309, 67046099030, 67046099130, 64725093003, 64725192403, 63629712501, 63629712502, 63629712503, 63629712504, 63629712505, 63629712506, 63629712507, 69189059101, 67046099630, 67046099730, 70518065200, 70518065201, 70518065202, 00054018813, 00054018913, 00093572056, 00093572156, 00228315403, 00228315473, 00228315503, 00228315573, 00378876793, 00378876893, 00406192303, 00406192403, 00406800503, 00406802003, 00781721664, 00781722764, 00781723864, 00781724964, 42291017430, 42291017530, 43598057930, 43598058030, 43598058130, 43598058230, 47781035503, 47781035603, 47781035703, 50268014415, 50268014515, 50383028793, 50383029493, 52427069203, 52427069403, 52427069803, 52427071203, 53217013830, 54569640800, 55700018430, 60429058630, 60429058730, 62175045232, 62175045832, 62756096964, 62756096983, 62756097064, 62756097083, 65162041503, 65162041509, 65162041603, 65162041609, 70518100700, 70518168400, 70518231100, 70518232700, 71335129601, 71335129602, 71335137801, 58284010014, 52440010014, 12496010001, 12496030001, 00490005100, 00490005130, 00490005160, 00490005190, 12496120201, 12496120203, 12496120401, 12496120403, 12496120801, 12496120803, 12496121201, 12496121203, 12496128302, 12496130602, 16590066605, 16590066630, 16590066705, 16590066730, 16590066790, 23490927003, 23490927006, 23490927009, 35356000407, 35356000430, 43063018407, 43063018430, 49999039507, 49999039515, 49999039530, 52959030430, 52959074930, 54569549600, 54569573900, 54569573901, 54569573902, 54569639900, 54868570700, 54868570701, 54868570702, 54868570703, 54868570704, 54868575000, 55045378403, 55700014730, 55887031204, 55887031215, 63629403401, 63629403402, 63629403403, 63874108403, 63874108503, 66336001530, 66336001630, 68071138003, 68071151003, 68258299903, 12496127802, 12496131002, 49999063830, 49999063930, 63629409201,

© 2022 Jones CM et al. *JAMA Psychiatry*.

	63874117303, 63874117403, 54123011430, 54123090730, 54123091430, 54123092930, 54123095730, 54123098630, 00228315567, 00378876716, 00378876816, 00781721606, 00781722706, 00781723806, 00781724906, 12496010002, 12496010005, 12496030002, 12496030005, 43063075306, 43598057901, 43598058001, 43598058101, 43598058201, 47781035511, 47781035611, 47781035711, 47781035811, 50090292400, 50268014411, 50268014511, 52427069211, 52427069411, 52427069811, 52427071211, 53217024630, 60429058611, 60429058633, 60429058711, 60429058733.
ER naltrexone from pharmacies	NDCs: 00406009203, 43063046915, 52125072702, 63459030042, 63629530402, 65757030001
Methadone from OTPs	HCPCS: G2067, G2078
Buprenorphine from OTPs	HCPCS: G2068, G2069, G2070, G2071, G2072, G2079
ER naltrexone from OTPs	HCPCS: G2073
**OUD and Behavioral Health-Related Services**	**ICD-10 CM Diagnosis Codes on Part A or B Service Claim**
OUD-Related Services	F1110, F1111, F11120, F11121x, F1114, F1115x, F1118x, F1119, F112x F1122x, F1123, F1124, F1125x, F1128x, F1129
Behavioral Health-Related Services	F060, F061, F062, F0630, F0631, F0632, F0633, F0634, F064, F068, F09, F1010, F10120, F10121, F10129, F1014, F10150, F10151, F10159, F10180, F10181, F10182, F10188, F1019, F1020, F10220, F10221, F10229, F10230, F10231, F10232, F10239, F1024, F10250, F10251, F10259, F1026, F1027, F10280, F10281, F10282, F10288, F1029, F10920, F10921, F10929, F1094, F10950, F10951, F10959, F1096, F1097, F10980, F10981, F10982, F10988, F1099, F1110, F11120, F11121, F11122, F11129, F1114, F11150, F11151, F11159, F11181, F11182, F11188, F1119, F1120, F11220, F11221, F11222, F11229, F1123, F1124, F11250, F11251, F11259, F11281, F11282, F11288, F1129, F1210, F12120, F12121, F12122, F12129, F12150, F12151, F12159, F12180, F12188, F1219, F1220, F12220, F12221, F12222, F12229, F12250, F12251, F12259, F12280, F12288, F1229, F1290, F12920, F12921, F12922, F12929, F12950, F12951, F12959, F12980, F12988, F1299, F1310, F13120, F13121, F13129, F1314, F13150, F13151, F13159, F13180, F13181, F13182, F13188, F1319, F1320, F13220, F13221, F13229, F13230, F13231, F13232, F13239, F1324, F13250, F13251, F13259, F1326, F1327, F13280, F13281, F13282, F13288, F1329, F1390, F13920, F13921, F13929, F13930, F13931, F13932, F13939, F1394, F13950, F13951, F13959, F1396, F1397, F13980, F13981, F13982, F13988, F1399, F1410, F14120, F14121, F14122, F14129, F1414, F14150, F14151, F14159, F14180, F14181, F14182, F14188, F1419, F1420, F14220, F14221, F14222, F14229, F1423, F1424, F14250, F14251, F14259, F14280, F14281, F14282, F14288, F1429, F1490, F14920, F14921, F14922, F14929, F1494, F14950, F14951, F14959, F14980, F14981, F14982, F14988, F1499, F1510, F15120, F15121, F15122, F15129, F1514, F15150, F15151, F15159, F15180, F15181, F15182, F15188, F1519, F1520, F15220, F15221, F15222, F15229, F1523, F1524, F15250, F15251, F15259, F15280, F15281, F15282, F15288, F1529, F1590, F15920, F15921, F15922, F15929, F1593, F1594, F15950, F15951, F15959, F15980, F15981, F15982, F15988, F1599, F1610, F16120, F16121, F16122, F16129, F1614, F16150, F16151, F16159, F16180, F16183, F16188, F1619, F1620, F16220, F16221, F16229, F1624, F16251, F16259, F16280, F16283, F16288, F1629, F1650, F1690, F16920, F16921, F16929, F1694, F16950, F16951, F16959, F16980, F16983, F16988, F1699, F17200, F17201, F17203, F17208, F17209, F17210, F17211, F17213, F17218, F17219, F17220, F17221, F17223, F17228, F17229, F17290, F17291, F17293, F17298, F17299, F1810, F18120, F18121, F18129, F1814, F18150, F18151, F18159, F1817, F18180, F18188, F1819, F1820, F18220, F18221, F18229, F1824, F18250, F18251, F18259, F1827, F18280, F18288, F1829, F1890, F18920, F18921, F18929, F1894, F18950, F18951, F18959, F1897, F18980, F18988, F1899, F1910, F19120, F19121, F19122, F19129, F1914, F19150, F19151, F19159, F1916, F1917, F19180, F19181, F19182, F19188, F1919, F1920, F19220, F19221, F19222, F19229, F19230, F19231, F19232, F19239, F1924, F19250, F19251, F19259, F1926, F1927, F19280, F19281, F19282, F19288, F1929, F1990, F19920, F19921, F19922, F19929, F19930, F19931, F19932, F19939, F1994, F19950, F19951, F19959, F1996, F1997, F19980, F19981, F19982, F19988, F1999, F200, F201, F202, F203, F205, F2081, F2089, F209, F21, F22, F23, F24, F250, F251, F258, F259, F28, F29, F3010, F3011, F3012, F3013, F302, F303, F304, F308, F309, F310, F3110, F3111, F3112, F3113, F312, F3130,

© 2022 Jones CM et al. *JAMA Psychiatry*.

	F3131, F3132, F314, F315, F3160, F3161, F3162, F3163, F3164, F3170, F3171, F3172, F3173, F3174, F3175, F3176, F3177, F3178, F3181, F3189, F319, F320, F321, F322, F323, F324, F325, F328, F3289, F329, F330, F331, F332, F333, F3340, F3341, F3342, F338, F339, F340, F341, F348, F3481, F3489, F349, F39, F4000, F4001, F4002, F4010, F4011, F40210, F40218, F40220, F40228, F40230, F40231, F40232, F40233, F40240, F40241, F40242, F40243, F40248, F40290, F40291, F40298, F408, F409, F410, F411, F413, F418, F419, F42, F422, F423, F424, F428, F429, F430, F4310, F4311, F4312, F4320, F4321, F4322, F4323, F4324, F4325, F4329, F438, F439, F440, F441, F442, F444, F445, F446, F447, F4481, F4489, F449, F450, F451, F4520, F4521, F4522, F4529, F4541, F4542, F458, F459, F488, F489, F5000, F5001, F5002, F502, F508, F509, F53, F54, F59, F600, F601, F602, F603, F604, F605, F606, F607, F6081, F6089, F609, F630, F631, F632, F633, F6381, F6389, F639, F641, F642, F648, F649, F6810, F6811, F6812, F6813, F688, F69, F900, F901, F902, F908, F909, F910, F911, F912, F913, F918, F919, F930, F938, F939, F983, F988, F989, F99
**Telehealth Services**	Only services that are on the Medicare telehealth services list are classified as telehealth eligible and those HCPCS codes billed with the POS = "02" or "95", "GT", "GQ", or "GO" modifiers are considered delivered via a telecommunication system and not in-person. Virtual check-ins were identified using HCPCS codes G2010, G2012 or G0071, as reported by the health care professional on the Part B Claim. E-visits were identified using the CPT codes 99421-99423 or HCPCS codes G2061-G2063, as reported by the rendering health care professional on the Part B Claim.
**Medically Treated Drug Overdose**	ICD-10 CM Diagnosis Codes: T36-T50
**Co-occurring conditions**	**ICD-10 CM Diagnosis Codes**
Substance use disorder	
Alcohol use disorder	F101X, F102X, F109X
Tobacco use disorder	F17
Cannabis use disorder	F121X, F122X, F129X
Cocaine use disorder	F141X, F142X, F149X
Sedative/hypnotic use disorder	F131X, F132X, F139X
Stimulant use disorder	F151X, F152X, F159X
Other psychoactive substance use disorder	F161X, F162X, F169X, F181X, F182X, F189X, F191X, F192X, F199X
Mental health diagnosis	**CMS Chronic Condition Warehouse (CCW) Flags: https://www2.ccwdata.org/web/guest/condition-categories**
Anxiety	Anxiety Disorders CCW Flag
Bipolar disorder	Bipolar Disorder CCW Flag
Major depression	Depression CCW Flag
Personality disorder	Personality Disorders CCW Flag
Attention deficit hyperactivity Disorder	ADHD, Conduct Disorders, and Hyperkinetic Syndrome CCW Flag
Post-traumatic stress disorder	Post-Traumatic Stress Disorder CCW Flag
Schizophrenia or other psychotic disorder	Schizophrenia and Other Psychotic Disorders CCW Flag
Chronic medical conditions	
Cancer	CCW Flags for: Breast Cancer, Colorectal Cancer, Endometrial Cancer, Lung Cancer, Prostate Cancer, Leukemias and Lymphomas
Diabetes	Diabetes CCW Flag
Cardiovascular and other circulatory	CCW Flags for: Atrial Fibrillation, Acute Myocardial Infarction, Ischemic Heart Disease, Heart Failure, Hypertension
Chronic respiratory disease	CCW Flags for: Asthma, Chronic Obstructive Pulmonary Disease, Bronchiectasis
Viral hepatitis	Viral Hepatitis CCW Flag
HIV	HIV/AIDS CCW Flag

© 2022 Jones CM et al. *JAMA Psychiatry*.

Obesity	Obesity CCW Flag
Liver disease, cirrhosis, and other liver conditions, excluding hepatitis	CCW Flags for: Liver Disease, Cirrhosis, and Other Liver Conditions (excluding Hepatitis)
Acute/chronic pain	CCW Flags for: Migraine and Chronic Headache, Fibromyalgia and Chronic Pain and Fatigue, Rheumatoid Arthritis/Osteoarthritis, Multiple Sclerosis and Transverse Myelitis, Sickle Cell Disease, Spinal Cord Injury, Pressure Ulcers and Chronic Ulcers

© 2022 Jones CM et al. *JAMA Psychiatry*.

eTable 2. Characteristics Associated with Receipt of OUD-Related Telehealth Services During Study Period Among Beneficiaries With OUD in the Pandemic Cohort by Dual Eligible Status

	**Dually Eligible Beneficiaries N=36,724**	**Non-Dually Eligible Beneficiaries N=33,773**
	**Adjusted ORs**[ǂ] **(95% CI)**	**Adjusted ORs**[ǂ] **(95% CI)**
**Receipt of MOUD During Study Period**		
No MOUD	Ref	Ref
MOUD from OTP only	**0.379 (0.323, 0.444)**	**0.270 (0.182, 0.400)**
MOUD from OTP and Pharmacy	**3.318 (2.342, 4.700)**	**4.835 (1.911, 12.235)**
ER Naltrexone from Pharmacy only	**1.646 (1.132, 2.393)**	0.633 (0.141, 2.842)
Buprenorphine from Pharmacy only	**4.115 (3.818, 4.437)**	**2.820 (2.547, 3.122)**
**Baseline Sex**		
Male	Ref	Ref
Female	**1.090 (1.028, 1.156)**	**1.097 (1.034, 1.164)**
**Baseline Age Group**		
18-44	Ref	Ref
45-64	0.966 (0.893, 1.044)	0.880 (0.729, 1.062)
65-74	0.993 (0.902, 1.093)	**0.798 (0.662, 0.963)**
75 or older	1.097 (0.974, 1.235)	**0.744 (0.613, 0.903)**
**Baseline Race/Ethnicity**		
Non-Hispanic White	Ref	Ref
Non-Hispanic African American	0.938 (0.863, 1.018)	0.917 (0.798, 1.053)
Non-Hispanic American Indian or Alaska Native	1.140 (0.912, 1.425)	0.853 (0.566, 1.286)
Non-Hispanic Asian or Pacific Islander	**1.460 (1.177, 1.810)**	0.791 (0.549, 1.140)
Non-Hispanic Other	1.180 (0.821, 1.697)	0.872 (0.599, 1.270)
Hispanic	1.037 (0.942, 1.142)	0.962 (0.818, 1.131)
**Baseline U.S Census Region**		
Northeast	Ref	Ref
Midwest	0.893 (0.673, 1.185)	0.731 (0.518, 1.033)
South	**0.662 (0.510, 0.858)**	**0.672 (0.488, 0.924)**
West	0.836 (0.632, 1.105)	0.877 (0.625, 1.232)
**Baseline County Urban-Rural Status**		
Metropolitan	Ref	Ref
Micropolitan	0.968 (0.900, 1.041)	0.938 (0.867, 1.015)
Rural	0.981 (0.817, 1.177)	0.898 (0.726, 1.112)
**Time Spent in Nursing Home from Baseline to End of Study**		
No time	Ref	Ref
Quantile 1	**0.608 (0.503, 0.734)**	**0.613 (0.506, 0.742)**
Quantile 2	**0.672 (0.554, 0.816)**	**0.671 (0.561, 0.802)**
Quantile 3	**0.572 (0.478, 0.684)**	**0.636 (0.519, 0.779)**
Quantile 4	**0.625 (0.543, 0.718)**	0.830 (0.576, 1.196)
**Baseline Other Substance Use Disorder Diagnosis***		

© 2022 Jones CM et al. *JAMA Psychiatry*.

Alcohol use disorder	0.957 (0.878, 1.042)	0.911 (0.813, 1.021)
Tobacco use disorder	**0.876 (0.824, 0.930)**	**0.915 (0.848, 0.987)**
Cannabis use disorder	**0.887 (0.805, 0.977)**	0.836 (0.697, 1.002)
Cocaine use disorder	1.000 (0.886, 1.129)	**0.670 (0.482, 0.932)**
Stimulant use disorder	0.908 (0.811, 1.016)	0.772 (0.591, 1.008)
Sedative/hypnotic disorder	**1.518 (1.374, 1.677)**	**1.604 (1.440, 1.787)**
Other psychoactive substance use disorder	**0.785 (0.720, 0.856)**	**0.748 (0.649, 0.863)**
**Baseline Mental Health Diagnosis***		
Anxiety	1.027 (0.962, 1.097)	0.948 (0.890, 1.009)
Bipolar disorder	1.030 (0.958, 1.108)	**1.190 (1.073, 1.319)**
Major depression	**1.091 (1.024, 1.161)**	**1.158 (1.088, 1.232)**
Personality disorder	0.923 (0.831, 1.026)	0.973 (0.836, 1.133)
Attention deficit hyperactivity disorder	1.088 (0.979, 1.210)	0.937 (0.786, 1.116)
Post-traumatic stress disorder	**1.227 (1.125, 1.338)**	0.985 (0.850, 1.141)
Schizophrenia or other psychotic disorder	**0.824 (0.755, 0.900)**	0.932 (0.794, 1.094)
**Baseline Other Chronic Medical Conditions***		
Cancer	**0.882 (0.796, 0.978)**	**0.889 (0.817, 0.966)**
Diabetes	**0.932 (0.875, 0.992)**	**0.926 (0.869, 0.986)**
Cardiovascular and other circulatory	**0.806 (0.752, 0.864)**	**0.881 (0.816, 0.951)**
Chronic respiratory disease	**0.870 (0.819, 0.924)**	**0.862 (0.808, 0.919)**
Viral hepatitis	**1.109 (1.013, 1.215)**	**1.175 (1.001, 1.379)**
HIV	1.171 (0.972, 1.411)	1.095 (0.737, 1.628)
Obesity	**1.090 (1.026, 1.159)**	0.979 (0.919, 1.042)
Liver disease cirrhosis and other liver conditions±	**0.765 (0.703, 0.831)**	**0.834 (0.758, 0.917)**
Acute/Chronic Pain	1.085 (0.999, 1.177)	1.029 (0.923, 1.148)
Bolded text represents statistically significant results		
ƗModels adjusted for all variables in the table		
* Reference group is not having condition		
± Excluding hepatitis		
To enable mutually exclusive groups for the regression analysis, 27 beneficiaries (0.30% of beneficiaries receiving MOUD) who received both buprenorphine and ER naltrexone from pharmacies during the study period were excluded from the analysis		

© 2022 Jones CM et al. *JAMA Psychiatry*.

eTable 3. Characteristics Associated With Receipt of Behavioral Health-Related Telehealth Services During Study Period Among Beneficiaries With OUD in the Pandemic Cohort by Dual Eligible Status

	Dually Eligible Beneficiaries N=36,724	Non-Dually Eligible Beneficiaries N=33,773
	Adjusted ORs[ǂ] (95% CI)	Adjusted ORs[ǂ] (95% CI)
**Receipt of MOUD During Study Period**		
No MOUD	Ref	Ref
MOUD from OTP only	**0.690 (0.622, 0.767)**	**0.542 (0.424, 0.694)**
MOUD from OTP and Pharmacy	1.341 (0.930, 1.933)	1.398 (0.516, 3.788)
ER Naltrexone from Pharmacy only	**1.445 (1.000, 2.088)**	3.032 (0.967, 9.504)
Buprenorphine from Pharmacy only	**2.016 (1.868, 2.177)**	**1.958 (1.769, 2.168)**
**Baseline Sex**		
Male	Ref	Ref
Female	**1.244 (1.185, 1.305)**	**1.193 (1.134, 1.256)**
**Baseline Age Group**		
18-44	Ref	Ref
45-64	1.013 (0.948, 1.083)	0.983 (0.830, 1.163)
65-74	0.926 (0.855, 1.004)	**0.811 (0.686, 0.960)**
75 or older	**0.801 (0.725, 0.885)**	**0.687 (0.578, 0.817)**
**Baseline Race/Ethnicity**		
Non-Hispanic White	Ref	Ref
Non-Hispanic African American	**0.911 (0.852, 0.973)**	**0.888 (0.789, 1.000)**
Non-Hispanic American Indian or Alaska Native	0.991 (0.819, 1.199)	1.013 (0.726, 1.412)
Non-Hispanic Asian or Pacific Islander	**1.294 (1.064, 1.575)**	0.888 (0.649, 1.215)
Non-Hispanic Other	1.145 (0.830, 1.580)	0.948 (0.695, 1.293)
Hispanic	0.994 (0.916, 1.079)	0.944 (0.818, 1.090)
**Baseline U.S Census Region**		
Northeast	Ref	Ref
Midwest	**0.762 (0.600, 0.968)**	**0.675 (0.514, 0.886)**
South	**0.620 (0.498, 0.771)**	**0.660 (0.514, 0.849)**
West	**0.786 (0.621, 0.994)**	0.811 (0.620, 1.060)
**Baseline County Urban-Rural Status**		
Metropolitan	Ref	Ref
Micropolitan	**0.895 (0.842, 0.950)**	**0.850 (0.794, 0.909)**
Rural	**0.853 (0.734, 0.992)**	**0.697 (0.582, 0.835)**
**Time Spent in Nursing Home from Baseline to End of Study**		
No time	Ref	Ref
Quantile 1	**0.580 (0.506, 0.664)**	**0.751 (0.652, 0.865)**
Quantile 2	**0.776 (0.674, 0.894)**	**0.862 (0.754, 0.985)**
Quantile 3	**0.806 (0.713, 0.910)**	0.891 (0.769, 1.033)
Quantile 4	**0.840 (0.761, 0.928)**	1.168 (0.882, 1.547)

© 2022 Jones CM et al. *JAMA Psychiatry*.

**Baseline Other Substance Use Disorder Diagnosis***		
Alcohol use disorder	**1.091 (1.017, 1.171)**	**1.103 (1.005, 1.210)**
Tobacco use disorder	**1.073 (1.021, 1.128)**	**1.069 (1.004, 1.139)**
Cannabis use disorder	0.951 (0.879, 1.030)	1.022 (0.881, 1.185)
Cocaine use disorder	0.922 (0.834, 1.019)	**0.611 (0.472, 0.791)**
Stimulant use disorder	**0.774 (0.705, 0.850)**	**0.747 (0.602, 0.926)**
Sedative/hypnotic disorder	**1.385 (1.266, 1.515)**	**1.457 (1.321, 1.608)**
Other psychoactive substance use disorder	**0.927 (0.864, 0.995)**	0.936 (0.835, 1.049)
**Baseline Mental Health Diagnosis***		
Anxiety	**1.599 (1.515, 1.686)**	**1.547 (1.467, 1.631)**
Bipolar disorder	**1.608 (1.514, 1.707)**	**1.840 (1.686, 2.009)**
Major depression	**1.903 (1.808, 2.003)**	**2.055 (1.949, 2.167)**
Personality disorder	**1.109 (1.016, 1.211)**	**1.434 (1.262, 1.629)**
Attention deficit hyperactivity disorder	**1.380 (1.257, 1.516)**	**1.585 (1.362, 1.845)**
Post-traumatic stress disorder	**1.553 (1.438, 1.678)**	**1.663 (1.463, 1.891)**
Schizophrenia or other psychotic disorder	**1.385 (1.291, 1.485)**	**1.499 (1.312, 1.711)**
**Baseline Other Chronic Medical Conditions***		
Cancer	**0.885 (0.816, 0.960)**	**0.928 (0.865, 0.995)**
Diabetes	0.978 (0.929, 1.023)	**0.937 (0.888, 0.989)**
Cardiovascular and other circulatory	**0.871 (0.820, 0.925)**	**0.922 (0.862, 0.987)**
Chronic respiratory disease	0.974 (0.927, 1.023)	0.951 (0.901, 1.004)
Viral hepatitis	**0.916 (0.850, 0.987)**	0.995 (0.867, 1.143)
HIV	**1.218 (1.043, 1.423)**	1.187 (0.845, 1.668)
Obesity	**1.108 (1.054, 1.167)**	1.005 (0.953, 1.060)
Liver disease cirrhosis and other liver conditions±	**0.866 (0.812, 0.924)**	**0.892 (0.825, 0.964)**
Acute/Chronic Pain	**1.097 (1.024, 1.177)**	**1.133 (1.028, 1.249)**
Bolded text represents statistically significant results		
ƗModels adjusted for all variables in the table		
* Reference group is not having condition		
± Excluding hepatitis		
To enable mutually exclusive groups for the regression analysis, 27 beneficiaries (0.30% of beneficiaries receiving MOUD) who received both buprenorphine and ER naltrexone from pharmacies during the study period were excluded from the analysis		

© 2022 Jones CM et al. *JAMA Psychiatry*.

eTable 4. Characteristics Associated With MOUD Retention for at Least 80% of Eligible Days During Study Period Among Beneficiaries With OUD in the Pandemic Cohort Receiving MOUD During the Study Period by Dual Eligible Status

	**Adjusted ORs[ǂ] (95% CI) Dually Eligible Beneficiaries N=6,441**	**Adjusted ORs[ǂ] (95% CI) Non-Dually Eligible Beneficiaries N=2,356**
**Receipt of OUD-Related Telehealth Service**	**1.281 (1.127, 1.455)**	**1.252 (1.024, 1.530)**
**Receipt of MOUD During Study Period**		
MOUD from OTP only	Ref	Ref
MOUD from OTP and Pharmacy	**0.394 (0.264, 0.589)**	**0.263 (0.072, 0.965)**
ER Naltrexone from Pharmacy only	**0.168 (0.095, 0.295)**	1.059 (0.324, 3.463)
Buprenorphine from Pharmacy only	**0.536 (0.468, 0.613)**	**0.775 (0.601, 0.998)**
**Baseline Sex**		
Male	Ref	Ref
Female	0.932 (0.833, 1.043)	0.840 (0.694, 1.018)
**Baseline Age Group**		
18-44	Ref	Ref
45-64	**1.151 (1.008, 1.315)**	**1.610 (1.134, 2.285)**
65-74	1.106 (0.920, 1.329)	1.276 (0.886, 1.839)
75 or older	1.174 (0.799, 1.727)	0.816 (0.521, 1.279)
**Baseline Race/Ethnicity**		
Non-Hispanic White	Ref	Ref
Non-Hispanic African American	**0.659 (0.549, 0.792)**	0.885 (0.577, 1.359)
Non-Hispanic American Indian or Alaska Native	0.957 (0.588, 1.557)	0.740 (0.294, 1.862)
Non-Hispanic Asian or Pacific Islander	0.918 (0.497, 1.696)	0.947 (0.266, 3.376)
Non-Hispanic Other	1.099 (0.453, 2.668)	0.146 (0.018, 1.164)
Hispanic	0.841 (0.681, 1.040)	0.713 (0.408, 1.245)
**Baseline U.S Census Region**		
Northeast	Ref	Ref
Midwest	0.914 (0.664, 1.258)	1.247 (0.901, 1.728)
South	0.953 (0.724, 1.253)	1.111 (0.842, 1.467)
West	**0.726 (0.531, 0.992)**	0.847 (0.628, 1.143)
**Baseline County Urban-Rural Status**		
Metropolitan	Ref	Ref
Micropolitan	0.973 (0.843, 1.124)	0.922 (0.727, 1.169)
Rural	1.100 (0.769, 1.576)	0.731 (0.359, 1.487)
**Time Spent in Nursing Home from Baseline to End of Study**		
No time	Ref	Ref
Quantile 1	**0.311 (0.155, 0.625)**	**0.367 (0.139, 0.966)**
Quantile 2	0.631 (0.354, 1.124)	0.453 (0.171, 1.203)
Quantile 3	**0.030 (0.004, 0.217)**	omitted

© 2022 Jones CM et al. *JAMA Psychiatry*.

Quantile 4	**0.267 (0.102, 0.698)**	omitted
**Baseline Other Substance Use Disorder Diagnosis***		
Alcohol use disorder	**0.716 (0.598, 0.858)**	0.785 (0.567, 1.087)
Tobacco use disorder	0.936 (0.833, 1.051)	1.038 (0.846, 1.275)
Cannabis use disorder	**0.753 (0.614, 0.924)**	0.815 (0.519, 1.279)
Cocaine use disorder	**0.579 (0.447, 0.750)**	0.534 (0.254, 1.123)
Stimulant use disorder	**0.550 (0.441, 0.687)**	0.635 (0.339, 1.189)
Sedative/hypnotic disorder	0.928 (0.728, 1.183)	0.765 (0.508, 1.151)
Other psychoactive substance use disorder	0.907 (0.780, 1.056)	**1.396 (1.036, 1.881)**
**Baseline Mental Health Diagnosis***		
Anxiety	0.902 (0.791, 1.028)	0.915 (0.745, 1.124)
Bipolar disorder	1.094 (0.948, 1.264)	0.816 (0.601, 1.108)
Major depression	0.899 (0.794, 1.018)	0.946 (0.770, 1.162)
Personality disorder	**0.618 (0.484, 0.789)**	1.109 (0.702, 1.751)
Attention deficit hyperactivity disorder	0.989 (0.805, 1.214)	**0.596 (0.363, 0.977)**
Post-traumatic stress disorder	1.062 (0.891, 1.267)	0.916 (0.609, 1.376)
Schizophrenia or other psychotic disorder	0.858 (0.712, 1.034)	**0.402 (0.235, 0.688)**
**Baseline Other Chronic Medical Conditions***		
Cancer	0.837 (0.623, 1.124)	**0.570 (0.394, 0.823)**
Diabetes	0.927 (0.804, 1.070)	0.897 (0.718, 1.120)
Cardiovascular and other circulatory	0.926 (0.814, 1.055)	**0.793 (0.640, 0.983)**
Chronic respiratory disease	**0.863 (0.756, 0.986)**	0.857 (0.682, 1.076)
Viral hepatitis	0.918 (0.786, 1.072)	0.713 (0.493, 1.030)
HIV	0.846 (0.579, 1.236)	**0.345 (0.123, 0.969)**
Obesity	1.116 (0.975, 1.278)	1.125 (0.905, 1.399)
Liver disease cirrhosis and other liver conditions±	0.839 (0.696, 1.011)	0.767 (0.553, 1.064)
Acute/Chronic Pain	**0.869 (0.766, 0.987)**	**0.721 (0.570, 0.913)**

Bolded text represents statistically significant results

ǂModels adjusted for all variables in the table

* Reference group is not having condition

± Excluding hepatitis

To enable mutually exclusive groups for the regression analysis, 27 beneficiaries (0.30% of beneficiaries receiving MOUD) who received both buprenorphine and ER naltrexone from pharmacies during the study period were excluded from the analysis

© 2022 Jones CM et al. *JAMA Psychiatry*.

eTable 5. Characteristics Associated With Experiencing Any Medically Treated Drug Overdose During Study Period Among Beneficiaries With OUD in the Pandemic Cohort by Dual Eligible Status

	**Adjusted ORs[ǂ] (95% CI) Dually Eligible Beneficiaries N=36,724**	**Adjusted ORs[ǂ] (95% CI) Non-Dually Eligible Beneficiaries N=33,773**
**Receipt of OUD-Related Telehealth Service**	**0.672 (0.622, 0.726)**	**0.674 (0.619, 0.734)**
**Receipt of MOUD During Study Period**		
No MOUD	Ref	Ref
MOUD from OTP only	**0.525 (0.446, 0.618)**	**0.530 (0.359, 0.783)**
MOUD from OTP and Pharmacy	1.409 (0.929, 2.137)	0.857 (0.235, 3.127)
ER Naltrexone from Pharmacy only	0.920 (0.626, 1.351)	0.711 (0.170, 2.972)
Buprenorphine from Pharmacy only	**0.901 (0.817, 0.994)**	0.889 (0.774, 1.021)
**Baseline Sex**		
Male	Ref	Ref
Female	**1.075 (1.013, 1.141)**	**1.071 (1.005, 1.142)**
**Baseline Age Group**		
18-44	Ref	Ref
45-64	**0.715 (0.660, 0.775)**	**0.726 (0.591, 0.893)**
65-74	**0.738 (0.669, 0.813)**	**0.720 (0.586, 0.885)**
75 or older	**0.656 (0.580, 0.741)**	**0.678 (0.548, 0.839)**
**Baseline Race/Ethnicity**		
Non-Hispanic White	Ref	Ref
Non-Hispanic African American	**1.120 (1.035, 1.211)**	0.906 (0.781, 1.051)
Non-Hispanic American Indian or Alaska Native	0.934 (0.741, 1.176)	1.374 (0.946, 1.997)
Non-Hispanic Asian or Pacific Islander	1.043 (0.813, 1.339)	**1.771 (1.252, 2.505)**
Non-Hispanic Other	1.014 (0.680, 1.511)	**1.670 (1.186, 2.351)**
Hispanic	0.913 (0.824, 1.012)	0.899 (0.743, 1.088)
**Baseline U.S Census Region**		
Northeast	Ref	Ref
Midwest	0.958 (0.825, 1.113)	1.071 (0.916, 1.252)
South	**0.844 (0.737, 0.967)**	0.920 (0.799, 1.060)
West	0.995 (0.856, 1.156)	1.087 (0.933, 1.269)
**Baseline County Urban-Rural Status**		
Metropolitan	Ref	Ref
Micropolitan	**0.886 (0.823, 0.953)**	0.948 (0.873, 1.030)
Rural	0.986 (0.819, 1.187)	1.087 (0.888, 1.331)
**Time Spent in Nursing Home from Baseline to End of Study**		
No time	Ref	Ref
Quantile 1	**1.662 (1.448, 1.906)**	**2.330 (2.030, 2.674)**
Quantile 2	**1.626 (1.403, 1.883)**	**1.698 (1.476, 1.954)**

© 2022 Jones CM et al. *JAMA Psychiatry*.

Quantile 3	**1.652 (1.455, 1.875)**	**2.180 (1.877, 2.532)**
Quantile 4	1.109 (0.988, 1.245)	1.153 (0.832, 1.597)
**Baseline Other Substance Use Disorder Diagnosis***		
Alcohol use disorder	**1.131 (1.047, 1.222)**	**1.286 (1.159, 1.428)**
Tobacco use disorder	**1.208 (1.136, 1.284)**	**1.149 (1.065, 1.240)**
Cannabis use disorder	**1.219 (1.119, 1.328)**	**1.311 (1.116, 1.539)**
Cocaine use disorder	**1.407 (1.269, 1.559)**	**1.666 (1.289, 2.155)**
Stimulant use disorder	**1.288 (1.166, 1.422)**	**1.440 (1.152, 1.800)**
Sedative/hypnotic disorder	**1.349 (1.225, 1.485)**	**1.254 (1.118, 1.407)**
Other psychoactive substance use disorder	**1.823 (1.690, 1.967)**	**1.712 (1.515, 1.934)**
**Baseline Mental Health Diagnosis***		
Anxiety	**1.338 (1.249, 1.434)**	**1.421 (1.327, 1.521)**
Bipolar disorder	**1.109 (1.034, 1.189)**	**1.237 (1.118, 1.369)**
Major depression	**1.261 (1.181, 1.347)**	**1.332 (1.244, 1.426)**
Personality disorder	**1.233 (1.124, 1.353)**	0.938 (0.807, 1.090)
Attention deficit hyperactivity disorder	1.040 (0.939, 1.152)	0.915 (0.764, 1.097)
Post-traumatic stress disorder	**0.905 (0.831, 0.986)**	0.985 (0.852, 1.140)
Schizophrenia or other psychotic disorder	**1.083 (1.002, 1.171)**	**1.397 (1.214, 1.608)**
**Baseline Other Chronic Medical Conditions***		
Cancer	**1.801 (1.651, 1.964)**	**1.993 (1.847, 2.151)**
Diabetes	**1.168 (1.099, 1.242)**	**1.154 (1.081, 1.232)**
Cardiovascular and other circulatory	**1.444 (1.335, 1.561)**	**1.516 (1.373, 1.675)**
Chronic respiratory disease	**1.299 (1.225, 1.378)**	**1.419 (1.331, 1.514)**
Viral hepatitis	1.044 (0.960, 1.136)	1.079 (0.922, 1.263)
HIV	0.964 (0.804, 1.157)	0.887 (0.578, 1.363)
Obesity	**1.063 (1.001, 1.129)**	1.058 (0.991, 1.130)
Liver disease cirrhosis and other liver conditions[±]	**1.538 (1.434, 1.650)**	**1.624 (1.494, 1.766)**
Acute/Chronic Pain	**1.233 (1.127, 1.349)**	**1.580 (1.370, 1.823)**

Bolded text represents statistically significant results

ƗModels adjusted for all variables in the table

* Reference group is not having condition

± Excluding hepatitis

To enable mutually exclusive groups for the regression analysis, 27 beneficiaries (0.30% of beneficiaries receiving MOUD) who received both buprenorphine and ER naltrexone from pharmacies during the study period were excluded from the analysis

© 2022 Jones CM et al. *JAMA Psychiatry*.
